# Hemodynamics study of a multilayer stent for the treatment of aneurysms

**DOI:** 10.1186/s12938-016-0248-0

**Published:** 2016-12-28

**Authors:** Yan Xiong, Xuhong Wang, Wentao Jiang, Xiaobao Tian, Qingyuan Wang, Yubo Fan, Yu Chen

**Affiliations:** 1 School of Manufacturing Science and Engineering, Sichuan University, 610065 Chengdu, China; 2 Department of Applied Mechanics, Sichuan University, 610065 Chengdu, China; 3 School of Biological Science and Medical Engineering, Beihang University, 100191 Beijing, China

**Keywords:** Multilayer stent, Aorticaneurysm, Blood flow, Pressure, WSS

## Abstract

**Background:**

The changes of hemodynamics caused by the implantation of multilayer stent (MS) have significant effects for aneurysm sac.

**Methods:**

Comparisons of 3D numerical models with/without a MS in an abdominal aortic aneurysm with a 90° branch vessel were numerically studied from the viewpoint of hemodynamics.

**Results:**

The results showed that: (1) The flow fields and Wall Shear Stress (WSS) are changed dramatically after MS implantation. The velocity of the blood flow in aneurysm sac decreases significantly and the regions of low-WSS increase. These help thrombus formation; (2) The pressure in aneurysm slightly decreases and keeps close to the normal level of blood pressure, however the risk of aneurysm enlargement or even rupture still exists; (3) The flux and the velocity in branch artery are reduced by about half after MS implantation. Due to the implantation of MS, the changes in the flow field causes the decrease of pressure/WSS in aneurysm sac and the blood flow in branch vessel.

**Conclusions:**

The implantation of MS into abdominal artery results in more low-WSS regions inside aneurysm which induces thrombus formation. The pressure is reduced slightly means the risk of aneurysm rupture exists.

## Background

An abdominal aortic aneurysm (AAA) is one of the common diseases which risk the lives of old people, its mortality rate reaches 75– 90% once AAA appears rupture. In USA 15,000 patients die each year because of the rupture of AAA and other complications such as stroke [1]. Multilayer stent (MS), unlike classical stents, is formed by a plurality of stabilized layers of biocompatible metal wires which are interlaced each other. MS is used to the treatment of visceral artery aneurysms (VAA) originally; it isolates aneurysm effectively and guarantees the supply of blood to the branches. Then MS is gradually used in the treatment of thoraco-abdominal aortic aneurysms (TAAA) in the complex and important branches [2, 3].

The renal artery aneurysm was first treatment with MS happened in 2008 in France [4], where a 78-year-old hypertensive man with complications was incidentally found to have a large saccular aneurysm in his renal artery involving the inferior renal artery, MS was implanted easily on the aneurysm neck covering the inferior renal artery, then the velocity of blood flow in the sac was quickly and dramatically decreased and blood pressure returned to the normal level. After 6 months, angiography showed that the aneurysm wall had shrunk completely and blood in the inferior renal artery branches returned to normal level. MS can overcome drawbacks which brought by covered stent (CS), providing a new idea in the treatment of AAA involving the branch artery. Polydorou et al. [5] reported that 9 patients with MS got 100% technical successes between December 2006 and November 2009; Fossaceca et al. [6] followed-up six patients who were treated by MSs for peripheral artery aneurysms from September 2010 to May 2011. Totally 12 stents were used, technical successes were achieved by 100%; Giampaolo et al. [2] reported that a 60-year-old man presenting a celiac trunk aneurysm refused open surgical repair and then was treated with a MS to reduce the strength of vortex and induce thrombosis, after 12 month it confirmed that the thrombosis was formed in the sac without impairment of the main branches. Ferrero et al. [3, 7] analyzed two males who had hepatic artery aneurysm were treated with MS, it showed the thrombosis of the aneurysmal sac and patency of the branches of hepatic artery by computed tomography (CT) scan in a 1 year follow-up. At present, MS is taken as a viable method to treat visceral artery aneurysms when patients are at high surgical risk. Claus et al. [8] reported that ten complex iliac artery aneurysm (IAA) were treated in 8 patients between October 2010 and August 2012, the results showed stent placement was technically successful and the mortality was 0%. Lowe et al. [9] reported 14 patients were treated with MS, 7 patients died including one fatal rupture; Vojko et al. [10] reported a 50-year-old man with two juxtarenal saccular aneurysms of abdominal aorta was treated with MS, and it showed the continuous shrinkage of the aneurysm sac in a 1 year follow-up. Alberto et al. [11]. implanted 5 MSs in 5 patients to treat hepatic artery aneurysms, 2 years follow-up showed technical successes; Morris et al. [12] treated visceral artery aneurysms (VAAs) in 5 patients with MSs and technical successes were achieved for all patients; Bouillot et al. [13] compared the process of intracranial aneurysms (IAs) treated with flow diverter stents through both PIV and CFD, the results showed there was a good PIV–CFD agreement. All above studies are related to the clinical aspects, but a few researches are about numerical simulation. Zhang et al. [14, 15] investigated overlapping BMS in the treatment of aortic aneurysm, TAWSS, OSI and RRT were compared and it was proved that the stents were effective and the blood flow velocity was decreased, but the pressure and low-WSS within the aneurysm sac, and blood supply for branch vessel were not considered.

Stent implantation causes the changes of intravascular hemodynamics, meanwhile the distribution of WSS and pressure on the vascular wall are changed. In this paper, we analyzed the flow fields, pressure, WSS in aneurysm with one branch artery in two situations: with and without MS. The results would provide some theoretical guidance for the optimization design for MS.

## Methods

### Models

Two computational models were established in this study. One is MS implantation in artery with one branch and the other without MS in the same situation.

**Fig. 1:**
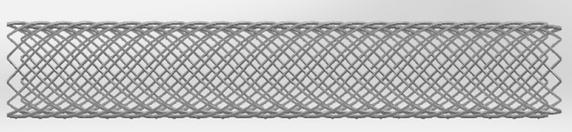
Multilayer stent model

The model of MS is based on US patent (Pub.No. US 2014/0180397 A1), as shown in Fig. 1. The length of MS is 40 mm and the diameter is 7 mm, and the cross-section of models is square (0.05 mm × 0.05 mm). The diameter of main artery is 7 mm and branch artery is 4 mm.

**Fig. 2:**
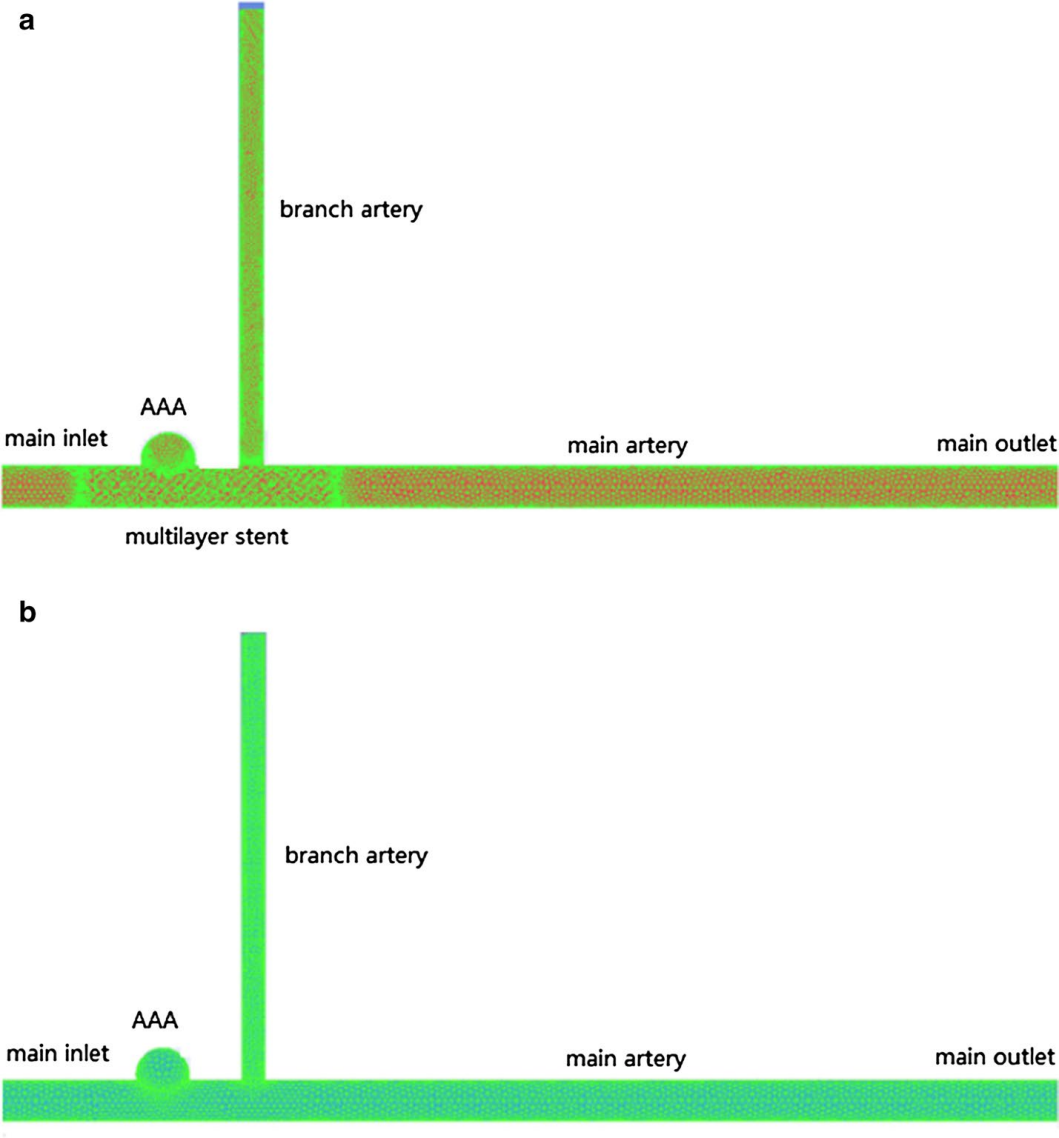
Two models **a** stented **b** unstented

The inlet velocity is 0.8 m/s, here was a 30-mm straight tubes at the inlet of the vascular model for the sake of making sure that the inlet flow was fully developed, and two straight tubes that the length 20 times of diameter at the outlet of main artery and branch artery respectively, as shown in Fig. 2.

**Fig. 3:**
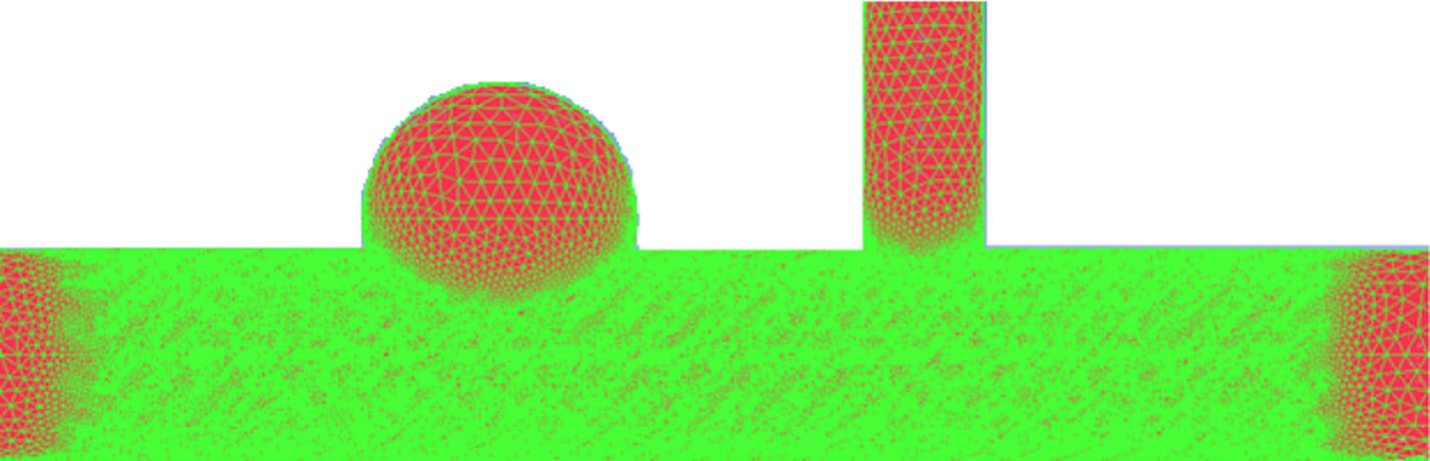
Mesh for MS model

The vascular models were established and meshed in the commercial pre-processing software (ICEM CFD 14.5, ANSYS Inc., Canonsburg, PA). In order to enhance the numerical accuracy, the refined grids near the surface of the stent and vessel were applied. The grid refinement stopped when the computational results did not change in terms of different grids. The number of the meshes in model A and model B was 12 million and 3 million, respectively (shown in Fig. 3).

### Boundary conditions

The assumption of incompressible Newtonian fluid was employed in this paper [16]. The blood flow was regarded as steady. Moreover, the assumption of the impermeable and rigid wall was applied in terms of the arterial wall. In comparison with the characteristics of the vascular models, the effects of wall elasticity and non-Newtonian behavior on blood flow could be neglected. Consequently, the continuity equation and Navier–Stokes equation are shown as follow.

1 $$\left\{ \begin{array}{l} \frac{{\partial u_{\text{j}} }}{{\partial x_{\text{j}} }} = 0 \quad ({\text{i, j}} = 1,2,3) \hfill \\ \rho \frac{{\partial u_{\text{j}} u_{\text{i}} }}{{\partial x_{\text{j}} }} = - \frac{\partial P}{{\partial x_{\text{i}} }} + \mu \frac{\partial }{{\partial x_{\text{j}} }}\left(\frac{{\partial u_{\text{i}} }}{{\partial x_{\text{j}} }} + \frac{{\partial u_{\text{j}} }}{{\partial x_{\text{i}} }}\right) \hfill \\ \end{array} \right.$$

where P and u are pressure and blood velocity, respectively. In the computational models, the density (ρ) and dynamic viscosity (μ) were set as 1055 kg/m^3^ and 3.5 × 10^−3^ kg/ms [11], respectively.

The distribution of blood velocity at the inlet was set as parabolic, and the maximum value was set as 0.8 m/s [13].

2 $$\left\{ {\begin{array}{l} {{\text{u}}_{\text{z}} |_{\text{inlet}} = {\text{u}}_{\text{m}} \times \left(1 - \frac{{{\text{x}}_{\text{i}}^{2} + {\text{x}}_{\text{j}}^{2} }}{{{\text{r}}^{2} }}\right)} \\ {{\text{u}}_{\text{r}} |_{\text{inlet}} = 0} \\ \end{array} } \right.$$

The axial and radial velocity (u_z_, u_r_) at the inlet were considered in the models, respectively. And the radius of the vessel is 3.5 mm. Moreover, the pressure at the outlet was set as zero.

3 $$\left. P \right|_{outlet} = 0$$

In this study, the computational models were established in a commercial CFD package, FLUENT (ANSYS Inc.), and the numerical analysis was carried out based on the finite volume method. In the computational settings, these models were set as 3D single-precision; the computations were conducted by segregated solver; SIMPLEC algorithm was used in the correction of velocity and pressure; the pressure discretization was conducted through using the standard format; and 2nd-order upwind was applied for solving the momentum equations. Moreover, these numerical simulations would converge when the residual value reached 0.0001.

## Results

### Flow fields

**Fig. 4:**
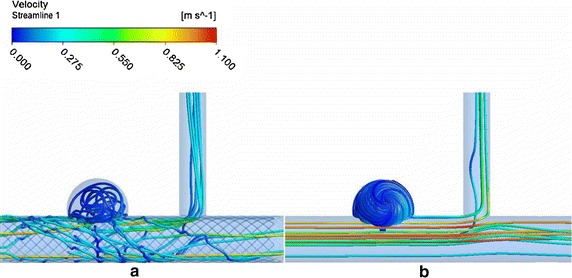
The flow fields in two models **a** stented **b** unstented

Streamlines for the two models are presented in Fig. 4, the flow field changes are obvious. The flow field is characterized by vortex in the unstented model and it means that there is recirculation in aneurysm sac. However after MS implantation, the vortex disappears; the flow is almost static in aneurysm sac. In the unstented situation, the flow field is stable and the streamlines are regular, after stent implantation, the flow velocity overall became slow and the streamlines display disorder.

The therapeutic action of MS is mainly a flow modulating effect, which changes the flow field and slows down the velocity of blood flow.

### Pressure and WSS

**Fig. 5:**
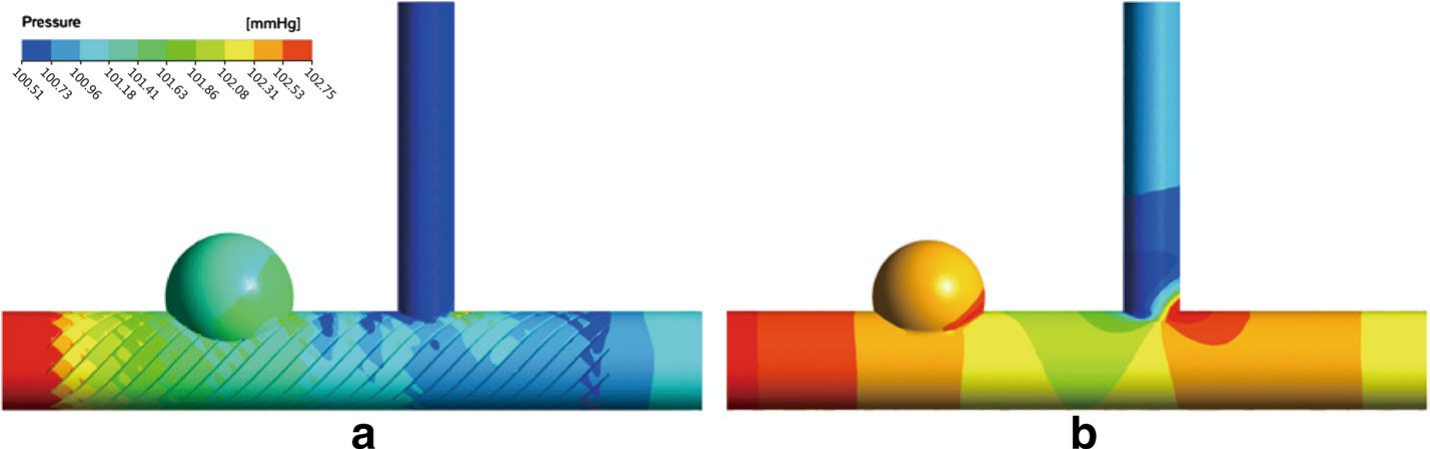
The distribution of pressure for two models **a** stented **b** unstented

The distributions of pressure for two models are presented in Fig. 5. After MS implantation, the values of pressure in aneurysm sac are decreased. When boundary conditions are set as 100 mmHg in the outlet of main artery and branch artery, the values of pressures are 100.36 vs. 100.58 mmHg in aneurysm sac and 999.83 vs. 100.21 mmHg in branch artery for stented/unstented models, respectively. The pressure changed slightly when MS implantation, a drop of pressure is not obvious.

**Fig. 6:**
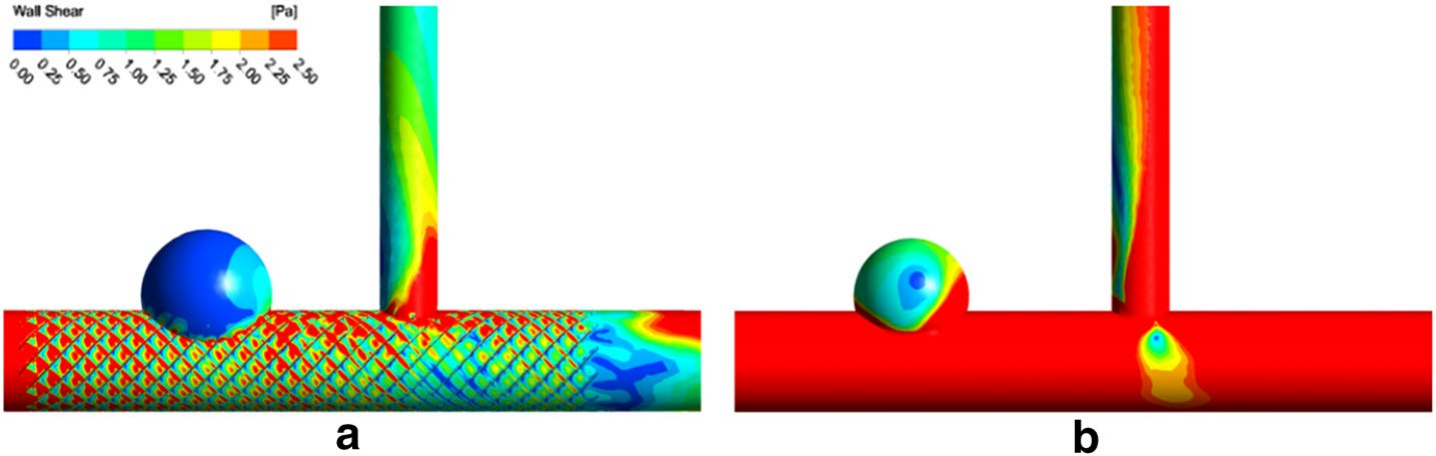
The distribution of WSS for two models **a** stented **b** unstented

The distributions of WSS for two models are presented in Fig. 6. The values of WSS on the wall of aneurysm are decreased significantly and most regions of the aneurysm walls are changed as the low-WSS regions After MS implantation. At the same time, the values of WSS on the branch artery reduced, and the low-WSS region appears in the upstream of intercross of main artery and branch artery. It needs to pay attention to the thrombus formation on branch artery except the aneurysm sac.

### Flux of branch vessel

**Fig. 7:**
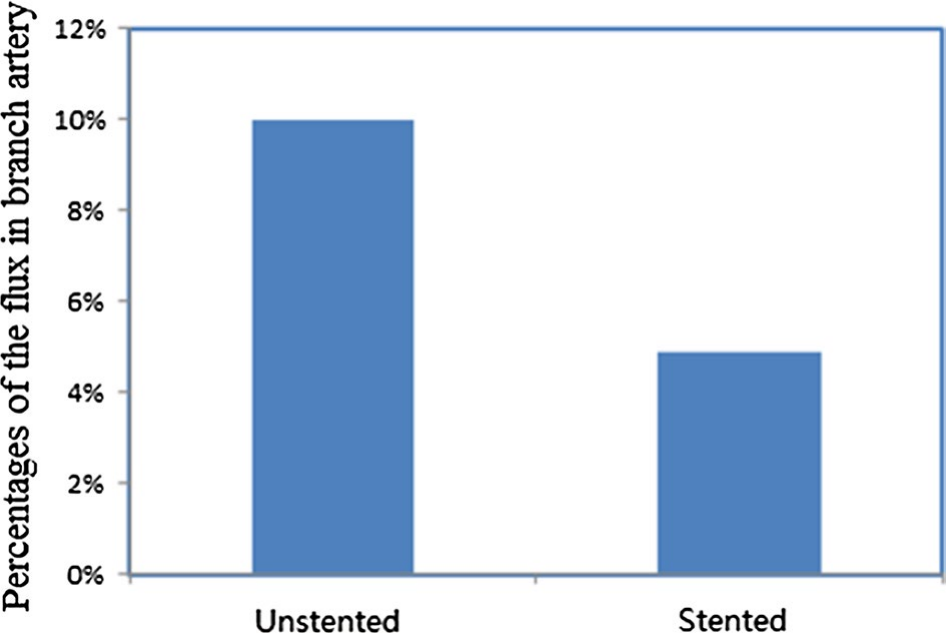
Percentage of the flux in branch artery to the total in two models

The percentages of the flux in branch artery to the total flux in the two models are shown in Fig. 7. The flux of the branch artery is only 10.0% of total flux in main and branch arteries. After MS implantation the flux in branch artery is reduced by half, with the percentage dropping to 4.9% of total flux. The velocity of branch artery decreased from 0.245 to 0.121 m/s. The branch artery has great influence on the supply of blood because it involves the kidney after MS implantation, which has a vital role in life activity. In order to have an effective function, it needs an adequate blood supply. An insufficient blood supply in renal artery would lead to refractory renovascular hypertension. Renovascular hypertension is renal arterial lesion which causes high blood pressure for involving kidney ischemia. It is consistent with any narrowing/blockage of blood supply to unilateral or bilateral renal arterial and branches. The increase of blood pressure caused by renal artery is proportional to the degree of stenosis of renal artery. A moderate stenosis would not lead to damage of the renal function and severe stenosis not only causes higher blood pressure, but also diffuses renal arteriole necrosis and causes renal insufficiency. These symptoms are similar to malignant hypertension observed in clinical practice. Whether or not a decline in the blood supply in the branch artery at a safe level is related to the organs involved in the branch artery. In the study the blood supply situations after MS implantation are numerically stimulated through CFD, and the results provide a support for clinics.

## Discussion

If an aneurysm is adjacent to or involves a major arterial branch, covered stent or graft would occlude the branch. MS, originally used in intracranial aneurysm repair, has been successful implanted for hepatic, renal, subclavian, iliac artery, type-B dissection and peripheral aneurysms. The multilayer structure has the effects of reducing and slowing the power of the vortices. The slowing of vortices and their alignment can create a filling that induces thrombosis [2].

In this study, the numerical results confirm that MS implantation leads to significant decrease of blood flow velocity and WSS in the aneurysm significantly, which make the low-WSS regions increase. MS decreased flow velocity and turbulent flow within aneurysm, the increase of low-WSS after MS implantation is a key factor for thrombus formation.

Hemodynamics is considered as one of the major reasons for aneurysm rupture. To understand the mechanisms of MS, it is necessary to analyze the changes of hemodynamics inside the sac for the two conditions: aneurysm absorption and rupture. In general, greater pressure and abnormal WSS are indications for aneurysm rupture. Low-WSS is recognized as thrombus formation, but an opposing option deems that ruptured aneurysms also have a greater portion of aneurysm under low-WSS [17]. Therefore, low-WSS in aneurysm should be studied more deeply and comprehensively.

The percentage of blood in branch is also an unknown factor. It is difficult to determine the influences of the reduction on branch organs when only 50% of blood supply in branch artery, but too much decrease of blood supply for branch organ is not a good choice. In this paper, we consider MS has only one layer, if multiple layer structure is applied, the decline of blood supply will be more severe. More layers result in a further decline of blood supply, therefore, after MS implantation blood supply of branch vessel is worth further studying, and at the same time we’ll consider the other commercial MS models in the next phase. The reduction of pressure in sac and the maintenance of blood supply in branch vessel are a contradiction, so the grid density of MS is the key factor to the treatment of aneurysm.

MS is considered as a perfect solution for treating complex aortic aneurysms because the blood flow inside the aneurysmal sac has not been isolated completely. It overcomes two main shortcomings brought by covered stent: one is unobstructed problem in branch artery, covered stent isolates arterial aneurysm and branch vessel at the same time, it would lead to some complications including paraplegia, cerebral blood deficiency and internal organ ischemia [18]; the other is that 30–50% patients with abdominal aortic aneurysms (AAAs) aren’t for endovascular repair because of short aneurysm neck etc. [19].The probability of aneurysm enlargement or rupture is still existed, because the pressure slightly decreased, the aneurysm wall is still under rather high pressure compared with the same situation of covered stent implantation. If AAA treated by MS with incomplete sac thrombosis, it can lead to further aneurysm enlargement and subsequence rupture [20, 21].

If MS is used as an alternative to traditional graft to the treatment of complex aortic aneurysms involving important branch vessel, we must think about the AAA re-enlargement risk. The endoleak is the most common problem of AAA rupture and it makes sac continue increasing and ultimately rupture. Theoretically, the MS may not seal the aneurysm, thus leading to rupture. The issue of blood flow resides inside the sac has not completely been solved, keeping the aneurysm enlargement and rupture probable [20]. But clinical experience showed that MS preserves the flow and excludes the aneurysm; the flow velocity outside the MS is then reduced up to 90% [22]. Follow-up after one year, and no type of endoleak was seen [23]. This problem still needs further observation and exploration as the evidences are limited to case reports and only a small number of patients with complex aortic aneurysm are followed-up.

Several assumptions are adopted in the study: It is a common assumption in numerical simulation that vascular wall is simplified as a rigid wall and there is little impact on the calculation results. Although the conditions are not completely true, what we consider are all in flow conditions, the elasticity of the surface is not particularly important. The arterial elasticity is reduced for atherosclerosis and the rigidity is strengthened for stent implantation, the assumptions are acceptable. The blood is simplified as an incompressible Newtonian fluid with constant density and viscosity. It is appropriate for large and medium arterial blood, because red blood cells are small relative to the blood vessel diameter in large artery, at the same time, shear rate of blood flow is very high in the vessel when the diameter is larger than 0.5 mm, therefore it is unnecessary to consider blood viscosity and shear rate. And when these parameters keep constant, the calculation error is less than 2%.

## Conclusions

In this paper, two models (with/without MS) are numerically studied and the influences of flow field, pressure and low WSS in the aneurysm sac are analyzed and discussed. The decrease of blood flow velocity resulted in more regions of low-WSS. This is good for thrombus formation, but the slight reduction of pressure makes the risk of aneurysm rupture still exist. In conclusion, numerical calculations and hemodynamics analysis help stent design optimization and make the doctor have a more intuitive understanding of the hemodynamic changes after MS implantation.

## References

[1] Desai M, Eaton-Evans J, Hillery C (2010). AAA stent-grafts: past problems and future prospects. Ann Biomed Eng.

[2] Carrafiello G, Rivolta N, Annoni M (2011). Endovascular repair of a celiac trunk aneurysm with a new multilayer stent. J Vasc Surg.

[3] Ferrero E, Ferri M, Viazzo A (2011). Endovascular treatment of hepatic artery aneurysm by multilayer stents: two cases and one-year follow-up. Interact CardioVasc Thorac Surg.

[4] Henry M, Polydorou A, Frid N (2008). Treatment of renal artery aneurysm with the multilayer stent. J Endovasc Ther.

[5] Polydorou A, Henry M, Bellenis I (2010). Endovascular treatment of aneurysm with side branches. A simple method. Myth or reality?. Hosp Chron.

[6] Fossaceca R, Guzzardi G, Stanca C (1994). Effectiveness of multilayer stent in the treatment of peripheral vascular diseases[J]. Plant Pathol.

[7] Ferrero E, Ferri M, Viazzo A (2011). Visceral artery aneurysms, an experience on 32 cases in a single center: treatment from surgery to multilayer stent. Ann Vasc Surg.

[8] Claus Christian P, Carsten M, Jens R (2013). Interventional exclusion of iliac artery aneurysms using the flow-diverting multilayer stent. Cardiovasc Interv Radiol.

[9] Lowe C, Worthington A, Serracino-Inglott F (2015). Repair of thoraco-abdominal and peri-renal aneurysms with the multi-layer flow-modulating stent: the UK pilot study. Eur J Vasc Endovasc Surg.

[10] Vojko F, Joze M, Silva B (2013). Treatment of primary infected juxtarenal aortic aneurysm with the multilayer stent. Vasc Endovasc Surg.

[11] Alberto B, Alberto A, Fulvio P (2013). Treatment of visceral aneurysm using multilayer stent: two-year follow-up results in five consecutive patients. Cardiovasc Interv Radiol.

[12] Morris L, Stefanov F, Hynes N (2015). An experimental evaluation of device/arterial wall compliance mismatch for four stent-graft devices and a multi-layer flow modulator device for the treatment of abdominal aortic aneurysms. Eur J Vasc Endovasc Surg.

[13] Bouillot P, Brina O, Ouared R (2015). Hemodynamic transition driven by stent porosity in sidewall aneurysms. J Biomech.

[14] Zhang P, Sun A, Zhan F (2014). Hemodynamic study of overlapping bare-metal stents intervention to aortic aneurysm. J Biomech.

[15] Zhang P, Liu X, Sun A (2015). Hemodynamic insight into overlapping bare-metal stents strategy in the treatment of aortic aneurysm. J Biomech.

[16] Zheng T, Wang W, Jiang WT, Deng XY, Fan YB (2012). Assessing hemodynamic performances of small diameter helical grafts: transient simulation. J Mech Med Biol.

[17] Jou L, Lee DH, Mawad M (2008). Wall shear stress on ruptured and unruptured intracranial aneurysms at the internal carotid artery. Am J Neuroradiol.

[18] Qiang LU, Yang J (2013). Development of multilayer aneurysm repair system for treatment of aortic aneurysm. Chin Heart J.

[19] Carpenter JP, Baum RA, Barker CF (2001). Impact of exclusion criteria on patient selection for endovascular abdominal aortic aneurysm repair. J Vasc Surg.

[20] Lazaris AM, Maheras AN, Vasdekis SN (2012). A multilayer stent in the aorta may not seal the aneurysm, thereby leading to rupture. J Vasc Surg.

[21] Geest JPV, Schmidt DE, Sacks MS (2008). The effects of anisotropy on the stress analyses of patient-specific abdominal aortic aneurysms. Ann Biomed Eng.

[22] Tolva VS, Bianchi PG, Cireni LV (2012). Multiple multilayer stents for thoracoabdominal aortic aneurysm: a possible new tool for aortic endovascular surgery. Int J Gen Med.

[23] de Vries JP (2012). Treatment of complex thoracoabdominal or juxtarenal aortic aneurysms with a multilayer stent. J Endovasc Ther.

